# Methylmercury, Amalgams, and Children’s Health: Björnberg et al. Respond

**Published:** 2006-03

**Authors:** Karolin Ask Björnberg, Marie Vahter, Gunilla Sandborgh Englund

**Affiliations:** Institute of Environmental Medicine, Karolinska Institutet, Stockholm, Sweden; Institute of Odontology, Karolinska Institutet, Huddinge, Sweden, E-mail: Gunilla.Sandborgh@ki.se

We acknowledge the points raised by Guzzi et al. regarding our recent publication on the transport of methylmercury and inorganic mercury to the fetus and breast-fed infant (Björnberg et al. 2005). The issue is whether the methylation of inorganic mercury from dental amalgam is of sufficient size to significantly contribute to the exposure to organic mercury.

Demethylation of methylmercury into inorganic mercury is the key step in the excretion process of methylmercury. This process occurs through microbial activity within the intestine. To a limited extent demethylation may also take place in the blood (Berglund et al. 2005). The kinetics of mercury in the human body may also include methylation of inorganic mercury, but the present knowledge of this process is rather limited. Based on findings from *in vitro* studies (Heintze et al. 1983; Lyttle et al. 1993), Guzzi et al. postulate that organic mercury in saliva is due to bacterial transformation in the oral cavity. It is of course of toxicologic interest to further investigate the biotransformation of mercury in both directions.

Inorganic mercury has been shown to accumulate in exocrine glands, and saliva is also one excretion pathway for inorganic mercury (Joselow 1968). It should be pointed out that the saliva samples used by Leistevuo et al. (2001), to which Guzzi et al. refer, consist of paraffin-stimulated whole saliva. Therefore it is not possible to ascertain to what extent the sample reflects excreted mercury from the central circulation (which could originate from both inorganic mercury and methylmercury exposure) or mercury derived directly from the fillings in the oral cavity.

In the study by Leistevuo et al. (2001), 15–18% of total mercury in saliva (5–12.5 nmol/L) was organic in a group of subjects with amalgam fillings. These subjects had, on average, 22 amalgam-filled surfaces (range, 2–51). In the non-amalgam group, the organic mercury was 2–5 nmol/L. As calculated by Guzzi et al., the subjects with amalgam would ingest about 2–3 μg/day of methylmercury derived from oral bacteria biomethylation of inorganic mercury.

Our study group of pregnant women (Björnberg et al. 2005) was exposed to low levels of both methylmercury and inorganic mercury, as reflected in the low concentrations found in blood. They consumed small amounts of fish and had few amalgam fillings, on average five amalgam-filled surfaces (range, 0–24). Therefore, the exposure to methylmercury possible originating from bacterial methylation of inorganic mercury in the oral cavity is far lower than that reported by Leistevuo et al. (2001).

It should also be pointed out that a meal of fish (200 g) containing 500 μg/kg methylmercury would result in the ingestion of 100 μg methylmercury. Also, consumption of fish with more moderate levels (50 μg/kg) would give rise to significant exposure (10 μg methylmercury).

Even though a small exposure to methylmercury may occur from bacterial methylation of inorganic mercury in the oral cavity, this exposure would be far lower than methylmercury exposure via fish consumption.
